# A Systematic Study of Lysine Succinylation in the Pathogenic Bacterium *Vibrio harveyi* in Aquatic Animals

**DOI:** 10.3390/molecules30112418

**Published:** 2025-05-31

**Authors:** Shuai Yang, Peng Zhou, Weijie Zhang, Yujia Zhang, Haiwei Guo, Yingzhu Wei, Xiaoxin Wen, Jichang Jian, Na Wang, Huanying Pang

**Affiliations:** 1 Fisheries College, Guangdong Ocean University, Zhanjiang 524025, China; yangshuai1@stu.gdou.edu.cn (S.Y.); 13211173241@stu.gdou.edu.cn (P.Z.); zhangweijie31@stu.gdou.edu.cn (W.Z.); zhangyujia11@stu.gdou.edu.cn (Y.Z.); haiweiguo976@gmail.com (H.G.); 13984858540@stu.gdou.edu.cn (Y.W.); czoteamo@163.com (X.W.); jianjc@gmail.com (J.J.); 2 Guangdong Provincial Key Laboratory of Aquatic Animal Disease Control and Healthy Culture, Zhanjiang 524025, China; 3 Chinese Academy of Quality and Inspection & Testing, Beijing 100176, China

**Keywords:** *Vibrio harveyi*, succinylation, virulence

## Abstract

*Vibrio harveyi,* a pathogenic vibrio, is ubiquitous and the most prevalent disease infecting tropical and subtropical mariculture animals in marine and estuarine environments. It presents a major risk to mariculture companies worldwide and can cause serious disease problems in aquaculture. Recent studies have shown that various pathogens employ post-translational modifications (PTMs) to regulate cellular processes. One of the major PTMs is lysine succinylation, which is widespread in eukaryotic and prokaryotic cells. Many basic biological functions of bacteria are associated with the regulation of lysine (K) succinylation (Ksuc). However, little is known about the role of lysine succinylation in *V. harveyi* pathogenesis. Here, we performed LC-MS/MS analysis of 1271 proteins from *V. harveyi* to identify 4252 Ksuc modification sites. The modification of S-ribosylhomocysteine lyase (LuxS) and transcription elongation factor GreA proteins by Ksuc was confirmed through immunoprecipitation combined with Western blot, further validating our proteomics results. Bioinformatics study revealed that the identified Ksuc proteins play roles in multiple cellular processes and vital metabolic pathways, including LuxS, biofilm exopolysaccharide biosynthesis protein EpsG, and the general secretory system (Sec systems), and are proteins that regulate bacterial virulence. Generally, this scientific study serves as the basis for additional research on the pathogenic nature of Ksuc in *V. harveyi* and reveals potential targets that would accelerate the manufacturing of attenuated vaccines.

## 1. Introduction

Post-translational modifications (PTMs) are critical regulatory mechanisms that play a pivotal role in a wide array of cellular processes, such as virulence, cellular metabolism, and gene expression in prokaryotic and eukaryotic cells [1]. During these processes, protein molecules can undergo the addition of simple chemical groups, including acetyl groups, hydroxyl, and methyl groups, as well as complex substances like lipids and sugars [2,3]. Various forms of PTMs have been linked to physiology and bacterial pathogenicity. Thus, solely determining the bacterial proteome alone may have limitations, and the characterization of the PTM is essential for a more comprehensive understanding of the fitness—in terms of bacterial pathogen resistance—and virulence [4]. There are 20 amino acid residues; lysine is commonly modified by various PTMs. For example, the Nε-acylation of proteins, which specifically targets the lysine residues, is a widely occurring PTM [5]. Recent decades have consistently demonstrated that lysine can undergo post-translational modification through various acylations [6]. Among hundreds of different PTMs, lysine residue acylation includes lysine 2-hydroxyisobutyrylation (Khib), lysine β-hydroxybutyrylation (Kbhb), lysine butyrylation (Kbu), lysine propionylation (Kpr), and lysine crotonylation (Kcr). Acylation on these lysine residues is a prerequisite that efficiently regulates proteins associated with biological processes [7].

Protein Ksuc is a relatively new reversible PTM that has been conserved in evolution. It catalyzes the transfer of the succinyl group of succinyl coenzyme A to a lysine residue in the protein fraction, forming succinyl lysine [8]. These changes in protein structure are accompanied by significant modifications in the physicochemical properties of the protein and its function [9]. In recent years, Ksuc proteins numbering in the thousands have been discovered in several kinds of bacteria and fungi, including *Candida albicans*, *Porphyromonas gingivalis*, and *Aeromonas hydrophila* [10,11,12]. Succinylation has been proposed to be more perturbing to protein properties compared with methylation and lactonization [13]. Most enzymes involved in controlling metabolic pathways, from important central metabolic pathways of bacteria to glycolysis, gluconeogenesis, TCA, and fatty acid metabolism, have been presented [14]. Additionally, many protein substrates have been identified, with Nε-succinylation controlling metabolic processes and cellular physiology [15]. PTMs may cause structural changes that favor significant alterations in protein function. Understanding cellular physiology and pathology requires the identification and analysis of protein succinylation modification sites, which may also yield crucial information for pertinent medication development and biomedical research. In the last two years, mass spectrometry and high-throughput techniques have been widely used to characterize Ksuc in a range of taxa, including bacteria and humans [16,17,18,19,20].

*V. harveyi* is a Gram-negative, halophilic bacterium with a rod-shaped or short-rod body that is a major bacterial pathogen in mollusks, fin-fish, and especially shrimp being intensively fed [21,22]. It is primarily distributed in marine and estuarine environments. Outbreaks of this pathogen result in serious mortality in marine species, leading to significant losses on a global scale. In addition, several scholars have reported strains of antibiotic-resistant bacteria in both aquaculture and clinics [23,24,25]. The systematic analysis and identification of the protein succinylation modification sites of *V. harveyi* are an indispensable reference for controlling the bacterium and preparing an attenuated vaccine. *V. harveyi* was one of the first bacteria shown to possess all three quorum-sensing systems: N-acylhomoserine lactone (AHL), Autoinducer-2 (AI-2), and Cholera autoinducer 1 (CAI-1), processes of signal transduction in the cell that are important regulatory mechanisms within the bacterium apprised alterations in the population or the community of bacteria around it based on the levels of particular autoinducers [26,27,28]. A threshold AI concentration triggers the activation of genes necessary for environmental adaptation, including the production of antibiotics, the type III secretion system, hemolysin, biofilm formation, and bacterial luminescence. Additionally, it secretes a number of virulence factors, including hemolysins, proteases, lipopolysaccharides (LPS), iron-binding capacity, interactions with phages, biofilm formation, and cytotoxins [29,30,31,32], all of which contribute to the pathogenic mechanism and need to be further understood.

Comprehensive Ksuc in various bacterial pathogens has underscored the significance of this PTM. Nevertheless, succinylated proteins in *V. harveyi* have not been thoroughly studied, as far as we are aware, representing a significant barrier to fully understanding the regulatory mechanisms of Ksuc in this pathogen. Therefore, we conducted the first extensive study to find Ksuc targets in *V. harveyi*. We explored Nε-succinylated PTMs using mass spectrometry and identified 4252 Ksuc sites on 1271 proteins of *V. harveyi*. In addition, bioinformatics analyses revealed the presence of numerous succinylated sites present on proteins involved in antibiotic biosynthesis and, to a lesser extent, purine metabolism, as well as showing different biological functions and processes involved in the biosynthesis of secondary and ribosomal metabolites. The findings of this study represent the first comprehensive Ksuc analysis of *V. harveyi* and, therefore, can be used for future studies on the biological function of Ksuc in *V. harveyi*.

## 2. Results and Discussion

### 2.1. Identification of Ksuc Peptides and Proteins in V. harveyi

**Figure 1:**
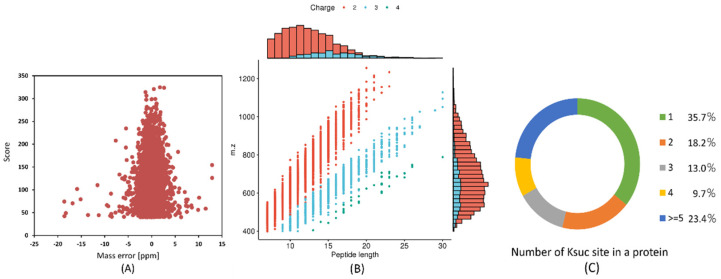
Profile of *V. harveyi* Ksuc proteome. (**A**) Mass error distributions for peptides with lysine succinylation. (**B**) Lysine succinylated peptide distribution based on length. (**C**) The pie chart illustrates the distribution of succinylation sites across different proteins.

We employed a combination of immunoaffinity enrichment of Ksuc peptides, utilizing highly specific succinylated antibodies, and LC-MS/MS to investigate succinylated proteins and peptides from *V. harveyi*. At an FDR threshold of 1% for peptides, a total of 4252 unique succinylated peptides, corresponding to 4252 succinylation sites, were identified across 1271 proteins in *V. harveyi* (Supplemental Table S1 and Table S2). The mass error for succinylated peptides varied between −20 and 15 ppm, suggesting that the MS dataset remained within the anticipated error range (Figure 1A). Peptides showed significant abundance based on their length, with the majority had 7–20 fragments (97.60%) and a few in the range of 20–30 lengths—approximately 2.40% of the peptides (Figure 1B). Additionally, among the 1271 succinylated proteins, 35.7% exhibited succinylation at a single site, while 18.2%, 13.0%, and 9.7% were modified at two, three, and four sites, respectively. Notably, 23.4% of the proteins displayed modifications at five or more sites (Figure 1C). In *V. harveyi*, DNA-directed RNA polymerase subunit beta RpoB was the most extensively succinylated protein (36 sites). Furthermore, 11 proteins exhibited a high number of succinylated sites (>15), including translation-associated proteins D0WUH1 (GlyS, 17), D0WY69 (IleS, 17), D0X0B8 (SecA, 17), D0WSJ3 (FusA, 27), D0WW35 (RpoC, 32), and D0WXG9 (ValS, 20); the major chaperone proteins D0WUB9 (DnaK, 19), glutamate dehydrogenase D0X0V8 (21), dihydrosulfanyl dehydrogenase D0WZ77 (LpdA, 17), anaerobic ribonucleoside-triphosphate reductase D0X1C5 (NrdD, 18). The high prevalence of Ksuc sites in the chaperone proteins identified in this research aligns with findings in cells, such as *Mycobacterium tuberculosis* and *Vibrio alginolyticus,* and warrants further study [14,33].

### 2.2. Functional Description of V. harveyi’s Lysine Succinylome

**Figure 2:**
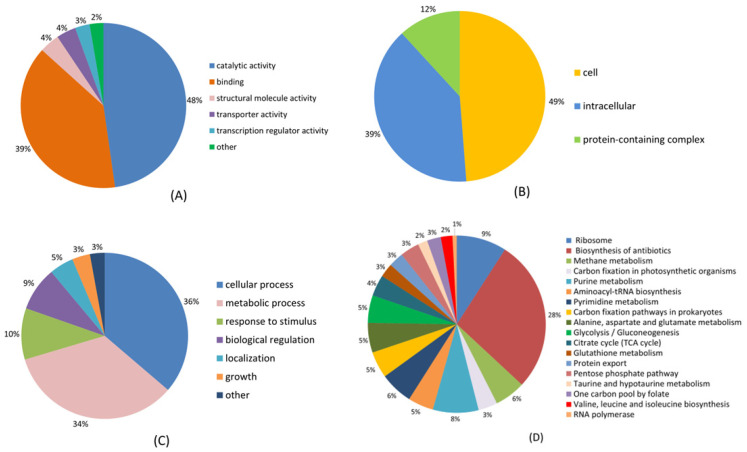
Functional classification of the identified succinylated proteins through GO functional classification and KEGG pathway analysis. (**A**) Molecular function. (**B**) Cell components. (**C**) Biological processes. (**D**) KEGG pathway analysis.

To examine the role of Ksuc, we conducted GO, KEGG, COG, and structural domain studies on every succinylated protein that was found. Classification results related to molecular function, cellular components, and the biological process revealed that the majority of succinylated proteins were linked to catalytic activity, cellular components, and cellular processes, constituting 48%, 49%, and 36% of the total succinylated proteins, respectively (Figure 2). In addition, other molecular functions, including binding, structural molecule activity, and transporter protein activity, constituted 39%, 4%, and 4% of the total identified proteins, respectively (Figure 2A), as well as intracellular complexes (39%) and protein-containing complexes (12%) by cellular composition (Figure 2B). In terms of biological processes, the other broad categories were proteins associated with metabolic processes (34%), response to stimuli (10%), and bioregulation (9%) (Figure 2C). GO analysis of succinylated particles showed that succinylated proteins are implicated in various molecular functions, cellular components, and biological processes and are closely associated with bacterial cellular activities.

KEGG analysis of succinylated proteins revealed that the majority of the identified proteins were predominantly involved in the biosynthesis of antibiotics (28%), ribosomes (9%), and purine metabolism (8%) (Figure 2D). In the current study, we identified 47 succinylated ribosomal proteins, of which 19 were from the 30 S ribosomal subunit and 28 were from the 50 S ribosomal subunit, all associated with translational processes. Notably, *M. tuberculosis*, *p. gingivalis,* and *E. coli* were also found to succinylate ribosomal proteins [33,34,35].

**Figure 3:**
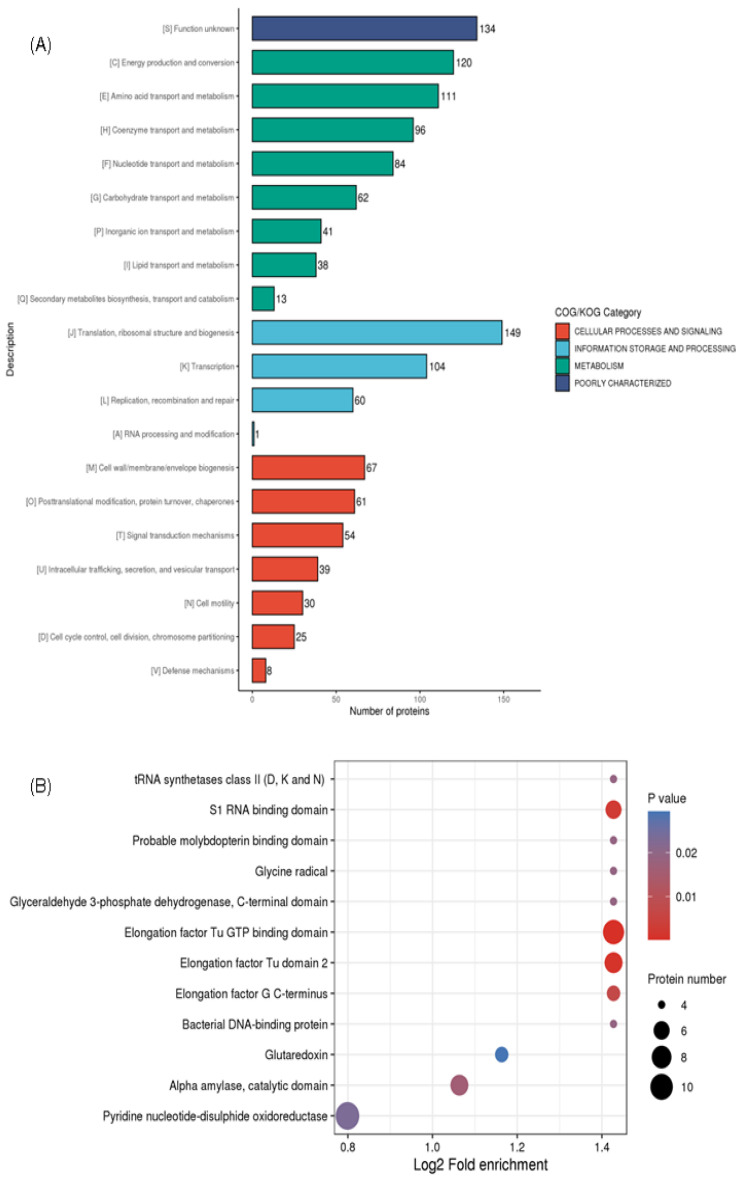
Functional annotation of the Ksuc in *V. harveyi*. (**A**) The COG analysis and (**B**) Bubble diagram depicting the enrichment analysis of structural domains in succinylated proteins (*p* < 0.05).

COG is a genome-scale analysis technique for studying the evolution and function of proteins. COG analysis identified significant categories, including translation, ribosome structure, and biogenesis (149) succinylated proteins, energy production and conversion (120), amino acid transport and metabolism (111), as well as post-cell wall/membrane/envelope biogenesis (67) (Figure 3A). Our findings aligned with earlier research on the succinyl proteome in *V. parahaemolyticus*, *Mycobacterium*, *M. tuberculosis*, and *E. coli* [33,34,36,37]. This implies that translated and metabolized proteins make up a large percentage of succinylated proteins.

This structural domain serves as the fundamental basis for the physiological functions of proteins. The domains of the identified succinylated proteins were annotated in order to further clarify the roles linked to succinylation. Figure 3B shows that the largest succinylated substrates with functional structural domains include alpha-amylase, S1 RNA binding domain, pyridine nucleotide-disulfide oxidoreductase, glutaredoxin, catalytic domain, and elongation factor Tu domain 2. Additionally, four tRNA synthetases class II (D, K, and N) and nine elongation factor Tu GTP binding domain proteins were found to undergo succinylation. This suggests that GTPase activity and protein synthesis may be regulated by succinylation alteration; thus, it coincides with similar succinylation modifications described in *A. hydrophila* [38].

### 2.3. Motif of Ksuc Peptides in V. harveyi

**Figure 4:**
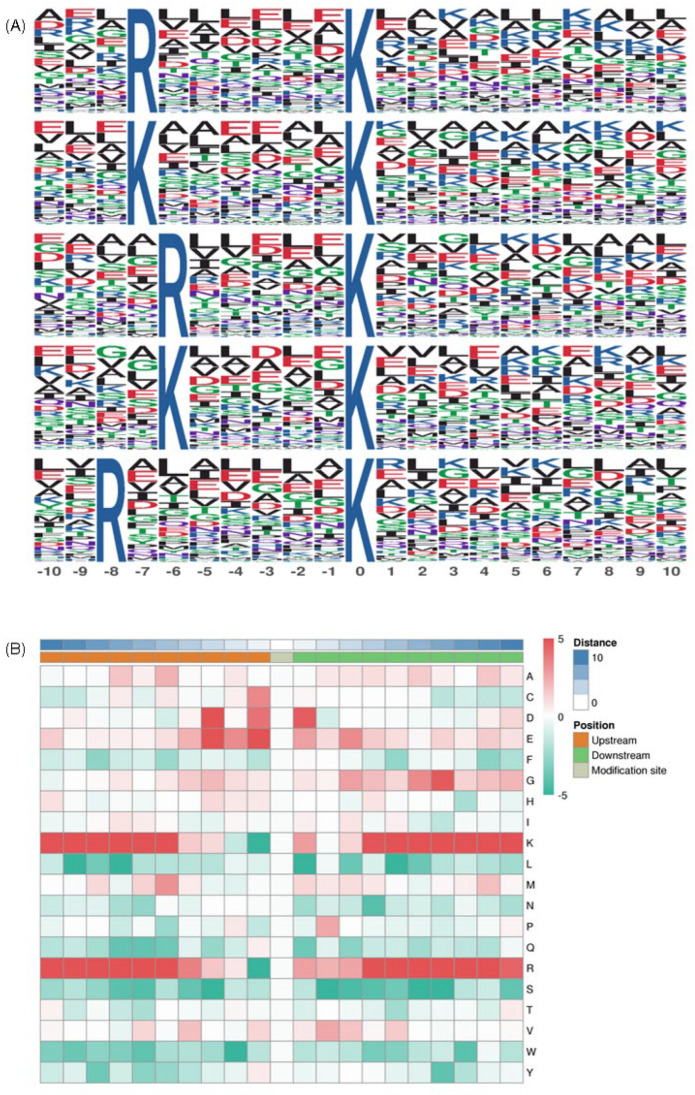
Motif analysis of Ksuc sites. (**A**) Sequence logos of motifs (*p* < 0.000001) were identified using MoMo software. (**B**) A heat map illustrating the distribution frequency of various types of amino acid residues surrounding Ksuc.

Next, we used the motif-X tool to further characterize the motifs of all succinylated peptides generated by *V. harveyi*, analyzing the sequences surrounding the lysine succinylation site, extending up to 10 amino acids on both sides, with a *p*-value threshold of <0.000001. As shown in Figure 4, five categories of motifs found in succinylation sites were matched: K******Ksuc, R*****Ksuc, K*****Ksuc, R*******, and R******Ksuc. An asterisk (*) denotes a random amino acid, and Ksuc is the succinylated lysine (Figure 4A). Furthermore, from the MoMo analysis, the amino acids around the modification site were visualized in a heat map to show changes in their frequency. Lysine was found most frequently at positions −5 to −10, 1, and 5 to 10; arginine at positions −4 to −10, 1, and 4 to 10; cysteine at position −1; aspartic acid at positions −1, −3, and 1; glutamic acid at positions −1 to −3; and glycine at position 7. On the contrary, the lowest frequencies were seen for lysine at position −1, leucine at −9, −7, 1, and 5, arginine at position −1, serine at −3, 2, 6, and 7, and tryptophan at position −2 (Figure 4B). The most favored sequence positions for lysine were −5 to −10 and 5 to 10, while for arginine, the favored sequences were −5 to −10 and 4 to 10. Overall, these results indicate that lysine residues and arginine residues occur with significant frequency near Ksuc.

### 2.4. Succinylation-Modified Proteases Are Widely Involved in Various Metabolic Pathways

**Figure 5:**
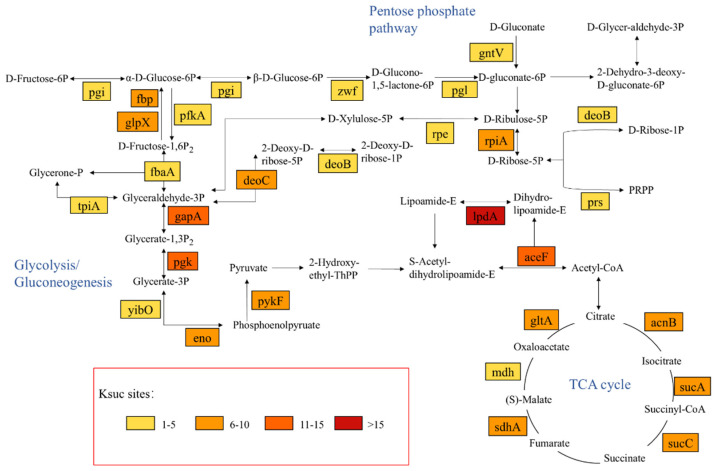
Schematic representation of Glycolysis/Gluconeogenesis, TCA cycle, and Pentose phosphate pathway with succinylation modifications. Staining of the identified succinylated proteins.

To investigate the potential functions of succinylation modifications, modifications of key molecules in specific critical biological pathways were analyzed. As shown in Figure 5, the succinylation modification site is present in the catalytic enzymes of many vital metabolic processes—especially in energy metabolism—such as Glycolysis/Gluconeogenesis, the TCA cycle, and the Pentose phosphate pathway, which are widely distributed. A total of 49 key metabolic enzymes undergo modification during these metabolic processes, and up to 10 or more sites were detected in 13 (26.5%) of these 49 proteins, which further implies that the occurrence of this modification is likely to impact metabolic regulation. All of these findings point to the critical function that lysine succinylation plays in *V. harveyi*’s Glycolysis/Gluconeogenesis, TCA cycle, and Pentose phosphate pathway. These results also imply that lysine succinylation may be conserved in *V. harveyi* and even in other prokaryotes because it is involved in a variety of regulatory mechanisms and may be involved in a variety of cellular activities.

### 2.5. Analysis of V. harveyi’s Succinylated Protein PPI Network

**Figure 6:**
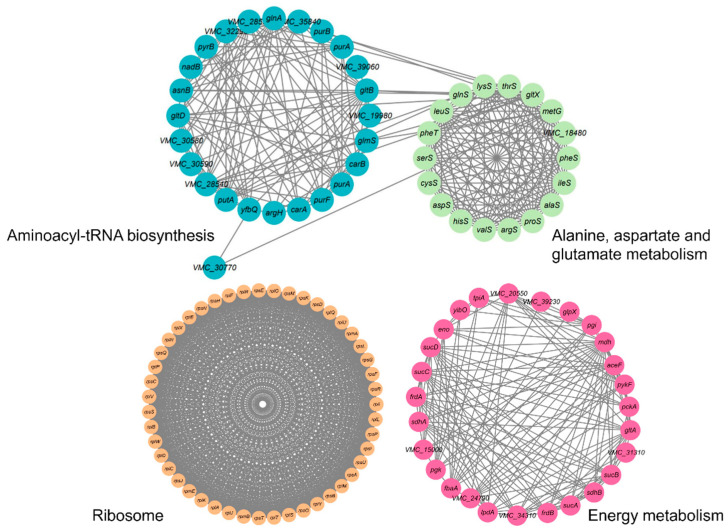
*V. harveyi*’s succinylated virulence factor PPI network.

PPIs are crucial for a variety of biological functions. The STRING software has been used for further exploration of metabolic pathways targeted by succinylation in *V. harveyi*. Our study identified 447 network nodes representing succinylated proteins and 6606 edges indicating protein interactions, thereby constructing the Ksuc network. The PPI network, generated using STRING, revealed 18 highly interconnected KEGG pathways enriched with succinylated proteins. Notably, the network highlighted clusters associated with energy metabolism, including the tricarboxylic acid (TCA) cycle and glycolysis, as well as alanine, aspartate, and glutamate metabolism, ribosomes, and aminoacyl-tRNA biosynthesis, as shown in Figure 6. The network composition in 47 succinylated ribosomal proteins and 24 succinylated proteins participating in aminoacyl-tRNA biosynthesis suggests that the Ksuc modifications play critical regulatory roles within these pathways, especially within protein translation. This agrees with the succinylation results obtained from *E. tarda*, *V. alginolyticus*, and *S. aureus* [14,39,40]. As such, based on the performed PPI network analysis of *V. harveyi*, its succinylated proteins established an intricate interaction network that forms a more substantial framework to further interpret their functional role within this pathogen.

### 2.6. Western Blot and Immunoprecipitation Are Used to Validate the GreA and LuxS Ksuc Proteins

**Figure 7:**
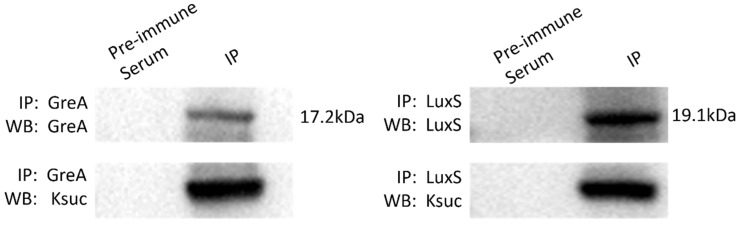
Validation of GreA and LuxS Ksuc proteins in *V. harveyi* using immunoprecipitation and Western blot. GreA and LuxS proteins were isolated via IP using specific antibodies, followed by Western blot with antibodies targeting GreA and LuxS (top panel) and anti-lysine succinylation antibodies (bottom panel).

Two proteins, GreA and LuxS, were examined using IP and Western blot in order to further validate the Ksuc. Western blotting with anti-succinylation and anti-target protein antibodies, respectively, was used for the detection of the GreA and LuxS proteins that were bound by their respective antibodies (Figure 7). Their respective antibodies captured GreA and LuxS proteins. The results showed that the corresponding antibodies indeed precipitated both GreA and LuxS proteins. In addition, the succinylation modifications of GreA and LuxS detected here were consistent with the Ksuc proteomics data, further supporting the validity of the proteomics results.

*V. harveyi* is a major aquaculture pathogen with a wide host range comprising fish, shrimp, and shellfish, responsible for serious economic losses in most areas worldwide [41]. Previous reports have evidenced that protein post-translational modifications, including phosphorylation, succinylation, and acetylation, are closely associated with bacterial virulence. Pathogenicity in *V. harveyi* is underlined by virulence factors (VFs); therefore, studies on these factors are of the utmost importance [42].

The online VFDB software analysis identified 21 succinylation sites across 10 proteins that influence the virulence of *V. harveyi* (Table 1). Ten succinylated proteins are involved in a variety of pathogenic processes, such as bacterial chemotaxis, the secretion system, and quorum sensing. Therefore, we hypothesized that succinylation may regulate bacterial virulence. Functional studies on bacterial chemotaxis, including *cheX*, *VMC_09770*, *cheW*, *cheV*, and *fliG*, have shown that bacterial chemotaxis plays a key role in allowing pathogens to enter the host [43]. The *epsG* gene, which is linked to the secretion system, plays a crucial role in bacterial infections. Interactions between *epsG* and the endosomal component *epsL* facilitate energy transfer from ATP hydrolysis to support secretion [44]. Proteins associated with quorum sensing include *yajC*, *luxS*, *luxO*, and *secB*; *luxS,* particularly, is an enzyme vital for AI-2 synthesis in the *V. harveyi* quorum sensing two-component system. Furthermore, in *Aeromonas hydrophila,* the synthesis of the quorum sensing autoinducer AI-2 is positively regulated by the succinylation of lysines on *luxS* at the K23 and K30 locations, which also modifies its competitiveness with other bacteria. The activated methyl cycle (AMC) maintains transmethylation capacity in all living cells. K30 succinylation on *luxS* may inhibit AMC [45]. In this study, the K23 and K29 sites of *luxS* were found to have Ksuc. *V. harveyi* should be investigated in depth to ascertain whether the site modification has the same function as *luxS* in *A*. *hydrophila*. Therefore, *V. harveyi’s* infection mechanism is closely tied to the combined effects of several virulence factors. The present investigation indicated that many virulence factors of *V. harveyi* are succinylated; thus, we postulate that Ksuc will play an indispensable role in controlling *V. harveyi’s* virulence.

## 3. Materials and Methods

### 3.1. Bacterial Strains and Protein Extraction

*V. harveyi* strain ZJ0603 was isolated from the spleen of a diseased fish, *Sillago sihama Forsskål*, at Guangdong Ocean University, Zhanjiang City, China [46]. This strain was cultured in thiosulfate citrate bile salts sucrose (TCBS) agar with an optimal incubation temperature of 28 °C. The strain was initially grown in TSB (add 2% NaCl) medium for 16–18 h to extract the protein. The strain was then subcultured the next day by inoculating the culture into a fresh TSB media (1:100 ratio) OD_600_ = 0.5 [47]. When the bacterial solution OD_600_ was approximately 1.0, bacterial precipitation was collected using centrifugation at 4 °C and 10,000 rpm for 5 min and washed twice with precooled PBS (0.01 M; pH = 7.4) buffer. The pellet was mechanically broken using sonication (power 45%, broken 6 s, interval 9 s) on ice for 15 min after being resuspended in PBS buffer. The protein was then separated from the supernatant by centrifuging the lysate. The BCA Protein Assay Kit (Beyotime, Shanghai, China) was used to determine the protein concentration [48].

### 3.2. Enrichment of Ksuc Peptides

Reduction and alkylation were performed using 10 mg protein samples, 10 mM DTT, and 20 mM IAA, as previously mentioned [49]. Trypsin at a ratio of 1:50 (*m*/*v*) was then used to enzymatically digest the sample for 16 h at 28 °C. During this step, agarose-conjugated anti-succinyl lysine antibodies (PTM Biolabs Inc., Hangzhou, China) were used to enrich Ksuc peptides by immunoaffinity chromatography (Supplemental Table S2) [50]. In brief, the digested peptides were treated with anti-succinyl lysine agarose beads for an entire night in NETN buffer. Following their elution with 1% trifluoroacetic acid (TFA), the enriched peptides were desalted using C18 ZipTips Millipore and subsequently delivered for MS identification as previously mentioned [13].

### 3.3. LC-MS/MS Analysis

The NanoElute Ultra Performance Liquid System was used to extract peptides that had been dissolved in liquid chromatography mobile phase A, which comprised 0.1% formic acid and 2% acetonitrile in water; 0.1% formic acid. Mobile phase B was 100% acetonitrile. The liquid phase gradient was set to 7–24%B for 0–40 min, 24–32%B for 40–52 min, 32–80%B for 52–56 min, and 80%B for 56–60 min, with a flow rate of 450 nL/min. TimsTOF Pro mass spectrometry was used to study the peptides after they were separated using an ultra-performance liquid chromatography system, injected into a Capillary ion source and ionized. A high-resolution TOF was used to identify and analyze the peptide precursor ion and its secondary fragments, with the ion source voltage set at 1.65 kV. The range of the secondary mass spectrum scan was 100–1700. The Parallel Cumulative Serial Fragmentation (PASEF) mode was used for data gathering. The dynamic exclusion period of tandem mass spectrometry was set to 30 s to prevent recurrent scanning of precursor ions, and a primary mass spectrometry was obtained following 10 PASEF modes to provide a secondary spectrum with precursor ion charge numbers in the range of 0–5 [51].

### 3.4. Data Processing

Proteome Discoverer 3.0 is a professional program for analyzing data from mass spectrometry. In accordance with the experimental strategy, the necessary analytical parameters were set after the raw mass spectrometry data were input into the database search software. In this investigation, secondary mass spectrometry data were retrieved using MaxQuant (v1.6.15.0). A shared contaminating library was added to the database to eliminate the impact of contaminating proteins on the identification findings, and a counter-database was introduced to compute the FDR resulting from random matching. The retrieval parameters were set as follows: *Vibrio_alginolyticus*_40B _674977_PR_20201110.fasta (4335 sequences) in the database; Digestion method: Trypsin. A maximum of five peptide modifications was permitted; the minimum peptide length was set at seven amino acid residues, and the number of missed cleavages was set at four. The main and fragment ion precursors were configured to have mass error tolerances of 20 ppm and 20 ppm, respectively; the mass error tolerance for fragment ions was 20 ppm. The variable modifications were methionine oxidation, protein N-terminal acetylation, and succinylation of lysine, while the fixed modification was carbamidomethyl (C) of cysteine alkylation. Proteins must include at least one unique peptide to be identified; the FDR for both PSM and protein identification was set at 1% (Figure 1).

### 3.5. Immunoprecipitation and Western Blot

Polyclonal antibodies specific to LuxS and GreA (Sangon, Shanghai, China), respectively, were used to precipitate target proteins. *V. harveyi* strain cell lysate was treated with LuxS and GreA antibodies for an entire night at 4 °C. Protein A/G beads that had been pre-washed three times with PBS solution were then added to the lysate and incubated for 10–14 h at 4 °C [52]. Beads were then centrifuged at 4 °C and washed five times with PBS buffer to elute the target proteins; 50 μL of SDS-PAGE Sample Loading Buffer (Beyotime, Shanghai, China) was added and boiled for 5 min, followed by SDS-PAGE and Western blot analyses [53].

Mouse monoclonal antibody to succinyl lysine (PTM Biolabs Inc., Hangzhou, China) and primary antibodies to LuxS (1:2000) and GreA (1:2000) were applied to the samples for two hours at 4 °C. Then, using a 1:10,000 dilution of TBST and 5% Western blotting blocking solution, the membrane was incubated with goat anti-mouse IgG (HL) conjugated with horseradish peroxidase (HRP) (Beyotime Biotechnology, Shanghai, China). Finally, the membrane was observed using the ECL system (Bio-Rad, Hercules, CA, USA), and the experimental data were photographed using the Tanon 5200 automatic chemiluminescence image analysis system [54].

### 3.6. Bioinformatics

We carried out a wide range of functional annotations for discovered proteins, including but not limited to GO, protein domain, KEGG pathway, and COG/KOG functional classification (Figure 2 and Figure 3), in order to be able to offer insights into the functional characteristics of various proteins. The identified succinylated proteins were annotated for KEGG pathway, molecular activities, biological processes, and GO keywords of cellular components using the internet application OmicsBean (http://www.omicsbean.cn/ accessed on 1 May 2024). The protein homology groups were defined by the COG database provided by NCBI (https://www.ncbi.nlm.nih.gov/COG/ accessed on 18 May 2024). Protein structural domains were annotated using STRING software (version 12.0). The online program MoMo (Modification Motifs, version 5.5.7 http://meme-suite.org/tools/momo?tdsourcetag=s_pcqq_aiomsg accessed on 1 May 2024) was used to evaluate amino acid sequence motifs [55]. GraphPad Prism 9.0 was used to create the pictures, and all statistical analyses were deemed significant when the adjusted *p*-value was less than 0.05. Protein–protein interactions (PPIs) were predicted by combining STRING with Cytoscape 3.10.0 software [56].

## 4. Conclusions

Certain fish and shrimp aquaculture industries are hampered in their development and growth by the opportunistic, salinophilic Gram-negative pathogen *V. harveyi*. Several investigations have shown that the succinylation of lysine residues in proteins is important for many biological processes and helps control bacterial physiology. This study identified 4252 succinylation sites that relate to 1271 proteins in *V. harveyi*. Furthermore, bioinformatics analysis indicated that the modification of proteins through Ksuc plays a pivotal regulatory role in various biological processes, including Carbon metabolism, the biosynthesis of secondary metabolites, microbial metabolism in different environments, energy metabolism, biosynthesis, and toxicity. Ksuc modifications of key virulence proteins can also affect virulence by altering bacterial life processes. In conclusion, this preliminary analysis of the succinic acidome of *V. harveyi* suggests potential biological functions of lysine-succinylated proteins. It may offer prospective targets for the development of attenuated vaccines.

## Figures and Tables

**Table 1:** Succinylated virulence factors in *V. harveyi*.

Protein	Gene Name	Ksuc Site	VF Class
D0WSF4	*cheX*	K13	Bacterial chemotaxis
D0WUX7	*VMC_09770*	K273, K46, K44, K229	Bacterial chemotaxis
D0WUX8	*cheV*	K39, K48	Bacterial chemotaxis
D0WVM2	*cheW*	K10	Bacterial chemotaxis
D0WVP7	*fliG*	K298	Bacterial chemotaxis
D0WSX5	*epsG*	K96	Type II secretion system
D0WU68	*yajC*	K48, K56, K69	Quorum sensing
D0WXB6	*luxS*	K23, K29	Quorum sensing
D0X2T3	*luxO*	K77, K193, K270, K277, K293	Quorum sensing
D0WYV3	*secB*	K43	Quorum sensing

## Data Availability

The manuscript contains all of the data produced during this research.

## Supplementary Materials

The following supporting information can be downloaded at: https://www.mdpi.com/article/10.3390/molecules30112418/s1, Table S1: List of succinylated proteins and their corresponding sites identified in *V. harveyi*. Table S2: List of succinylated peptides identified in *V. harveyi*.
